# Patterns of screen use and their association with sleep among medical students: a cross-sectional study

**DOI:** 10.3389/fpubh.2026.1936680

**Published:** 2026-09-18

**Authors:** Dolina Gencheva, Fedya Nikolov

**Affiliations:** 1 First Department of Internal Diseases, Medical University of Plovdiv, Plovdiv, Bulgaria; 2 Clinic of Cardiology, University Multi-Profile Hospital for Active Treatment “Sveti Georgi”, Plovdiv, Bulgaria

**Keywords:** daytime sleep, digital health, medical students, nighttime sleep, screen time, screen use, sleep, sleep patterns

## Abstract

**Background:**

Screen use and reduced sleep have become an integral part of medical education and daily life, raising concerns regarding the potential impact on health. This study investigated screen time patterns, sleep characteristics, and their associations among medical students.

**Aim:**

To assess daily screen time, nocturnal and daytime sleep patterns, and perceived adverse effects of screen use among Bulgarian and foreign medical students, and to investigate the associations between screen time and certain sleep characteristics.

**Methods:**

A cross-sectional questionnaire-based study was conducted among 1,063 medical students. Data on daily screen time, purpose of screen use, perceived adverse effects, nocturnal sleep duration, bedtime, daytime sleep, and lifestyle characteristics were collected. Group comparisons were performed according to student status (Bulgarian vs. foreign) and sex, and correlation analyses were used to assess associations between screen time and sleep-related parameters.

**Results:**

Mean daily screen time was 5.64 ± 2.81 h/day, with foreign students reporting significantly longer exposure than Bulgarian students (6.61 ± 3.06 vs. 4.79 ± 2.25 h/day; *p* < 0.001). Entertainment (78.3%) and studying/scientific activities (72.5%) were the most common purposes of screen use. Mean nocturnal sleep duration was 6.68 ± 1.18 h; 42.0% of students slept < 7 h/night, while 40.8% reported a usual bedtime after midnight. Overall, 55.3% reported daytime sleep, of whom 61.9% had naps lasting >30 min. Greater screen time was consistently, but weakly associated with later bedtime, whereas its association with nocturnal sleep duration was weak and limited to selected subgroups. Screen time also had a weak positive association with daytime sleep duration.

**Conclusions:**

Prolonged screen exposure and suboptimal sleep patterns are common among medical students. Greater screen time appears to be more strongly associated with delayed sleep timing than reduced sleep duration. Promoting healthier screen use habits and sleep hygiene are necessary.

## Introduction

Screen time is defined as the summed amount of time an individual spends using screen-based electronic devices, including smartphones, tablets, computers, televisions, and video game consoles (1). Due to the rapid digitalization of the modern world, screen-based technologies have now become an almost obligatory part of daily life and are widely used in education, work, communication, and entertainment. As a result, screen time comprises a broad spectrum of both passive activities, such as watching television or streaming media, and interactive activities, including internet surfing, social media use, gaming, studying, and other forms of digital engagement (2). Harvey et al. analyzed data from over 2 million adults aged 16–64 years across 46 countries to examine global trends in screen use between 2012 and 2019. They reported a worrying rise in overall daily screen exposure, with average screen time increasing from approximately 9 h per day in 2012 to around 11 h in 2019. This growth was attributable to the increased use of mobile devices, online platforms, and gaming devices, reflecting a transition from traditional forms of media consumption, such as television, toward more interactive and digitally connected activities. The highest use was among young people between 16 and 34 (3).

Increasing screen exposure has become a growing concern due to its potential implications for psychological wellbeing. Evidence from the OECD, based on data from 14 countries, suggests that individuals reporting higher levels of personal screen use are more likely to experience poorer subjective wellbeing. Specifically spending more than 5 h daily on screens for non-essential activities has been associated with a higher likelihood of reduced mental wellbeing, lower life satisfaction, and a diminished sense of purpose. These relationships appear to be influenced by other lifestyle-related factors, as inadequate sleep, lower levels of physical activity, and social isolation may further contribute to negative mental health outcomes among individuals with high screen exposure (2).

Beyond its effects on psychological health, there is emerging information linking screen time to adverse physical health outcomes, especially in the field of cardiovascular and metabolic health. Excessive screen time has been associated with several adverse physical health outcomes, primarily mediated by sedentary behavior and lifestyle displacement. A systematic review of reviews by Stiglic and Viner (33) found evidence linking higher screen time to increased adiposity and obesity risk among children and adolescents. The authors also reported associations between greater screen exposure and higher energy intake and poorer quality of diet, suggesting that prolonged screen use may contribute to unhealthy eating patterns. Furthermore, higher levels of screen-based sedentary behavior may displace opportunities for physical activity and have been associated with lower cardiorespiratory fitness (4). In addition, prolonged screen exposure may adversely affect sleep duration, while insufficient sleep has been associated with an increased risk of obesity and metabolic disturbances (5, 6).

A 2024 systematic review and meta-analysis of 19 prospective cohort studies involving more than 1.47 million participants found that higher sedentary time was associated with increased risks of fatal and non-fatal cardiovascular disease, with the associations remaining evident across different levels of sedentary exposure. Moreover, replacing sedentary time with light-intensity physical activity was associated with a lower risk of cardiovascular events (7). In a large prospective cohort study of 143,180 adults, Joundi et al. demonstrated that excessive leisure sedentary time was associated with a markedly increased risk of stroke among individuals younger than 60 years who had low levels of physical activity. Participants reporting ≥8 h of leisure sedentary time per day had more than a four-fold higher risk of stroke compared with those with lower sedentary time and higher physical activity levels (adjusted HR 4.50; 95% CI 1.64–12.3), (8). Sleep represents another essential, yet often overlooked, component of cardiovascular and metabolic health. Alongside physical activity and diet, adequate sleep contributes to the maintenance of physiological balance and regulation of multiple biological processes. A recent umbrella review by Yang et al. (9), incorporating 14 systematic reviews comprising 23 meta-analyses and 11 Mendelian randomization studies, found that short sleep duration was associated with increased risks of obesity, type 2 diabetes, hypertension, stroke, and coronary heart disease. Mendelian randomization analyses further supported potential causal relationships between short sleep and obesity, hypertension, and coronary heart disease. Long sleep duration was also associated with several adverse cardiometabolic outcomes in observational studies (9).

Medical students represent a relevant population for studying screen time and sleep behaviors, as their intense academic schedules, prolonged periods of studying, and high workload may contribute to an increased reliance on digital devices and insufficient sleep. The combination of extensive screen exposure for both educational and recreational purposes, irregular daily routines, and academic pressure may negatively influence sleep duration and quality. Therefore, assessing these behaviors among medical students may provide important insights into lifestyle patterns within this population and help identify opportunities for targeted interventions aimed at promoting healthier screen use and sleep habits.

At the Medical University of Plovdiv, Medicine is a six-year full-time programme, consisting of 10 semesters followed by a 12-month pre-diploma clinical clerkship. The curriculum is structured according to the European Credit Transfer and Accumulation System (ECTS), with the programme progressing from foundational and pre-clinical disciplines in the earlier years toward increasingly intensive clinical education in the later years (10). This structure is broadly consistent with medical education across Europe. European Union legislation requires basic medical training to comprise at least 6 years of study or 5,500 h of theoretical and practical training (11). Similarly, 6-year, 360-ECTS medical programmes are implemented at medical schools in several European countries, including Italy, Greece, and Portugal (12–14).

## Aim

The aim of this study was to assess daily screen time, nighttime and daytime sleep patterns, and perceived adverse effects of screen use among Bulgarian and foreign medical students and to investigate the associations between screen time and selected sleep characteristics, including sleep duration, bedtime, and daytime sleep. The study further sought to address the limited evidence available on these characteristics among medical students in Bulgaria and to explore whether screen-use and sleep patterns differ between Bulgarian and foreign students studying within the same university environment.

## Materials and methods

### Study design

We conducted a cross-sectional questionnaire-based study among medical students of the Medical University of Plovdiv, a public medical university in Bulgaria. The questionnaire was developed specifically for this study based on the study objectives and relevant literature on screen use and sleep among medical students. It assessed screen time, sleep patterns, and perceived effects of screen use and was not intended as a psychometric scale. Participants were recruited using a non-probability convenience sampling approach with voluntary participation. Data was collected between May and December 2024 using an anonymous online questionnaire created in Microsoft Forms (Microsoft Corporation, Albuquerque, NM, USA). Access to the survey was restricted to institutional university accounts, and the “one response per person” setting was enabled to prevent duplicate submissions and ensure participation exclusively by enrolled students. Participation was voluntary and anonymous. No personally identifiable information, including names or e-mail addresses, was collected. Participants were required to confirm all of the following inclusion criteria before accessing the survey: to be at least 18 years of age, currently enrolled as medical students at the Medical University of Plovdiv, and to provide electronic informed consent for the use of their anonymized responses for research purposes. Exclusion criteria: Individuals who did not meet the eligibility criteria or did not provide electronic informed consent were not allowed access to the form. The survey comprised items on sociodemographic characteristics, screen time, sleep habits, and other lifestyle factors relevant to health, such as smoking and alcohol use. The survey included dichotomous questions, multiple-choice items, and open-ended numerical responses. Data were reviewed for internal consistency, and questionnaires containing implausible or contradictory responses were excluded from the corresponding analyses. The survey was disseminated through the university's Microsoft Teams platform, during lectures and practical classes, and via QR code posters placed in commonly visited locations across the university campus, including libraries and student facilities. Identical Bulgarian- and English-language versions of the questionnaire were available to Bulgarian and foreign students, respectively. The study protocol was approved by the Ethics Committee of the Medical University of Plovdiv (Protocol No. 3/21.03.2024). *A priori* sample size calculation was performed based on the total population of 2,884 medical students enrolled at the Medical University of Plovdiv. Assuming a 95% confidence level, a 3% margin of error, and a conservative expected proportion of 50%, the minimum required sample size was 780 participants. The final sample of 1,063 students exceeded this requirement, corresponding to an overall response rate of approximately 37%.

### Statistical analysis

Statistical analyses were performed using IBM SPSS Statistics version 27.0 (IBM Corp., Armonk, NY, USA) and MedCalc version 19.6.3 (MedCalc Software, Mariakerke, Belgium). Statistical significance was defined as a two-sided *p*-value < 0.05. Continuous variables were summarized using appropriate descriptive statistics according to data distribution, while categorical variables were presented as frequencies and percentages. Normality was assessed using the Kolmogorov–Smirnov and Shapiro–Wilk tests. Comparisons between groups were performed using the independent-samples *t*-test or the Mann–Whitney *U* test, as appropriate. Associations between categorical variables were evaluated using the Chi-square test, Fisher's exact test, or the Fisher–Freeman–Halton exact test. Correlations between screen time and sleep-related variables were assessed using Spearman's rank correlation coefficient. Potential outliers and extreme observations were identified using boxplot analysis in SPSS (Explore procedure). These observations were subsequently assessed for plausibility and internal consistency, and statistical extremeness alone was not considered sufficient grounds for exclusion. In addition, we performed a sensitivity analysis based on the achieved sample size. With *n* = 1,063, α = 0.05, two-sided testing, and 80% power, the study could detect correlations of approximately *r* = 0.086.

## Results

### Sociodemographic characteristics of the study population

The study included 1,063 medical students, of whom 646 (60.8%) were women and 417 (39.2%) were men. Based on their reported country of permanent residence, participants were categorized into two groups: Bulgarian students, comprising those who indicated Bulgaria as their permanent residence (*n* = 563; 53.0%), and foreign students, defined as those reporting another country of residence (*n* = 500; 47.0%). The mean age of the participants was 21.97 ± 2.49 years, with an age range of 18–37 years. The Bulgarian and foreign student groups did not differ significantly in terms of age (21.85 ± 2.39 vs. 22.12 ± 2.59 years, *p* = 0.055) or sex distribution (39.6% vs. 38.8% male, *p* = 0.801). The proportions of Bulgarian and foreign students did not differ significantly in the first year (20.2% vs. 19.2%, *p* = 0.683), fourth year (30.7% vs. 27.8%, *p* = 0.300), fifth year (11.2% vs. 12.4%, *p* = 0.545), or sixth year (11.0% vs. 14.0%, *p* = 0.139), whereas Bulgarian students were more in the second year (17.8% vs. 12.0%, *p* = 0.008) and foreign students - in the third year (14.6% vs. 9.1%, *p* = 0.005). Among students who reported Bulgaria as their country of permanent residence, the majority identified as Bulgarian (85.3%), while Turkish (10.3%) and German (1.8%) were the next most frequently reported ethnicities. Among foreign students, Pakistani ethnicity was most commonly reported (14.2%), followed by Greek (13.6%) and Italian (11.6%). More details about the sociodemographic characteristics of the participants are available elsewhere (15). Regarding lifestyle-related characteristics, 62.9% of participants reported alcohol consumption, whereas 37.1% did not consume alcohol. In terms of smoking status, 19.7% were active smokers and 4.7% were former smokers, while 75.6% were never-smokers. Based on the recommended physical activity thresholds (≥150 min of moderate-intensity or ≥75 min of vigorous-intensity aerobic activity per week), 64.3% of participants were classified as physically active. Regarding employment status, 20.9% of participants were employed, while 79.1% were not.

### Screen time duration

Screen time duration data was available for 1,057 participants (99.4%). The mean daily screen time was 5.64 ± 2.81 h, ranging from 1 to 18 h. The distribution of screen time was significantly non-normal (Kolmogorov–Smirnov test, *p* < 0.001), with a mode of 5 h/day. No statistically significant association was identified between screen time and age or between screen time and year of study (Spearman's correlation, *p* > 0.05). Foreign students reported significantly longer daily screen time than Bulgarian students (6.61 ± 3.06 vs. 4.79 ± 2.25 h, *p* < 0.001). This difference remained significant in both male (6.73 ± 3.06 vs. 4.99 ± 2.30 h, *p* < 0.001) and female students (6.53 ± 3.06 vs. 4.65 ± 2.20 h, *p* < 0.001). No significant sex differences in mean screen time were observed (*p* = 0.088 for Bulgarian students and *p* = 0.370 for foreign students), Supplementary Table 1. The most frequently reported category of daily screen time was 2–4 h (37.2%), followed by 4.01–6 h (31.9%) and 6.01–8 h (16.0%). Only 1.6% of participants reported < 2 h of daily screen time, whereas 5.5% reported more than 10 h/day. Details in Table 1.

**Table 1:** Frequency distribution of answers to the question “How many hours is your screen time per day?”^*^

Screen time (hours)	*n*	%	Sp
2–4	395	37.2	1.5
4.01–6	339	31.9	1.4
6.01–8	170	16.0	1.1
8.01–10	78	7.3	0.8
Above 10	58	5.5	0.7
Below 2	17	1.6	0.4
No data	6	0.6	0.2
**Total number**	1,063	100.0	

^*^Valid daily screen-time data were available for 1,057 of 1,063 participants.

The distribution of screen time differed significantly between Bulgarian and foreign students, Table 2. The majority of the Bulgarian students reported 2–4 h/day, which was more than twice as much compared to the foreign students in that category (49.9% vs. 23.1%, *p* < 0.001), whereas most of the foreign students (32.0%) reported spending between 4.01 and 6 h in front of a screen. Moreover, they were also more likely, compared to Bulgarian students to spend prolonged screen time of 6.01–8 h (22.5% vs. 10.5%, *p* < 0.001), 8.01–10 h (11.9% vs. 3.4%, *p* < 0.001), and more than 10 h/day (9.1% vs. 2.3%, *p* < 0.001). Similar differences were observed among both male and female students in the groups. No statistically significant differences in screen time categories were identified when comparing between the two sexes, Supplementary Table 2.

**Table 2:** Comparison of daily screen time categories between Bulgarian and foreign medical students, overall and stratified by sex.^*^

Group	Screen time (hours)	Bulgarian students		Foreign students		*p*
		*n*	%	*n*	%	
All (*n* = 1,057)	Below 2	10	1.8	7	1.4	0.607
	2–4	281	49.9	114	23.1	**< 0.001**
	4.01–6	181	32.1	158	32.0	0.972
	6.01–8	59	10.5	111	22.5	**< 0.001**
	8.01–10	19	3.4	59	11.9	**< 0.001**
	Above 10	13	2.3	45	9.1	**< 0.001**
Men (*n* = 415)	Below 2	4	1.8	3	1.6	0.944
	2–4	105	47.1	41	21.4	**< 0.001**
	4.01–6	69	30.9	60	31.3	0.970
	6.01–8	30	13.5	47	24.5	**0.004**
	8.01–10	8	3.6	22	11.5	**0.002**
	Above 10	7	3.1	19	9.9	**0.004**
Women (*n* = 642)	Below 2	6	1.8	4	1.3	0.611
	2–4	176	51.8	73	24.2	**< 0.001**
	4.01–6	112	32.9	98	32.5	0.914
	6.01–8	29	8.5	64	21.2	**< 0.001**
	8.01–10	11	3.2	37	12.3	**< 0.001**
	Above 10	6	1.8	26	8.6	**0.015**

^*^Valid daily screen-time data were available for 1,057 of 1,063 participants. The bold values indicate statistically significant *p*-values.

### Primary purpose of screen use

Participants were asked, “Most of your screen time is spent on which two of the following?” (up to two answers permitted), Table 3. The most frequently selected response was “Entertainment” (78.3%), followed by “Studying/scientific process” (72.5%), “Seeking information for non-entertainment purposes, but not related to a studying/scientific process or work” (18.8%), and “Work” (7.5%). Compared with Bulgarian students, more foreign students selected “Studying/scientific process” (76.6% vs. 68.9%, *p* = 0.005) and “Work” (9.6% vs. 5.7%, *p* = 0.016), whereas Bulgarian students more often selected “Seeking information for non-entertainment purposes, but not related to a studying/scientific process or work” (21.8% vs. 15.4%, *p* = 0.008). Among female students, “Entertainment” was selected significantly more frequently by Bulgarian women than by foreign ones (82.4% vs. 75.8%, *p* = 0.039). Sex-based comparisons showed that women, more frequently than men, selected “Studying/scientific process” as one of the two activities that accounted for most of their screen time (77.2% vs. 65.2%, *p* < 0.001). Men, on the other hand, compared to women, more frequently selected “Work” (10.8% vs. 5.4%, *p* = 0.001) and “Seeking information for non-entertainment purposes, but not related to a studying/scientific process or work” (22.1% vs. 16.7%, *p* = 0.028). Among Bulgarian students, these sex differences remained significant for all three response categories. In contrast, among foreign students, only “Studying/scientific process” was selected significantly more frequently by women than by men (80.7% vs. 70.1%, *p* = 0.006).

**Table 3:** Comparative analysis of the two groups of students and the two sexes according to the answers to the question “Most of your screen time is spent on which two of the following?”

Group	Answers	Bulgarian students		Foreign students		*p*
		*n*	%	*n*	%	
All (*n* = 1,063)	Studying/scientific process	388	68.9	383	76.6	**0.005**
	Work	32	5.7	48	9.6	**0.016**
	Seeking information for non-entertainment purposes, but not related to a studying/scientific process or work	123	21.8	77	15.4	**0.008**
	Entertainment	451	80.1	381	76.2	0.124
Men (*n* = 417)	Studying/scientific process	136	61.0	136	70.1	0.052
	Work	23	10.3	22	11.3	0.743
	Seeking information for non-entertainment purposes, but not related to a studying/scientific process or work	59	26.5	33	17.0	**0.020**
	Entertainment	171	76.7	149	76.8	0.981
Women (*n* = 646)	Studying/scientific process	252	74.1	247	80.7	**0.046**
	Work	9	2.6	26	8.5	**< 0.001**
	Seeking information for non-entertainment purposes, but not related to a studying/scientific process or work	64	18.8	44	14.4	0.135
	Entertainment	280	82.4	232	75.8	**0.039**
Group	Answers	Men		Women		*p*
		*n*	%	*n*	%	
All (*n* = 1,063)	Studying/scientific process	272	65.2	499	77.2	**< 0.001**
	Work	45	10.8	35	5.4	**0.001**
	Seeking information for non-entertainment purposes, but not related to a studying/scientific process or work	92	22.1	108	16.7	**0.028**
	Entertainment	320	76.7	512	79.3	0.316
Bulgarian students (*n* = 563)	Studying/scientific process	136	61.0	252	74.1	**< 0.001**
	Work	23	10.3	9	2.6	**< 0.001**
	Seeking information for non-entertainment purposes, but not related to a studying/scientific process or work	59	26.5	64	18.8	**0.031**
	Entertainment	171	76.7	280	82.4	0.098
Foreign students (*n* = 500)	Studying/scientific process	136	70.1	247	80.7	**0.006**
	Work	22	11.3	26	8.5	0.300
	Seeking information for non-entertainment purposes, but not related to a studying/scientific process or work	33	17.0	44	14.4	0.433
	Entertainment	149	76.8	232	75.8	0.798

The bold values indicate statistically significant *p*-values.

### Perception of negative effects

Participants most commonly perceived that excessive screen time negatively affected their sleep (66.9%), eyesight (55.7%), mental state (38.6%), and time available for physical activity (36.8%). Fewer participants reported negative effects on face-to-face social interactions (26.4%), household responsibilities (23.9%), family time (21.9%), or work (16.7%), while only 3.9% reported no adverse effects, details in Table 4. Bulgarian students more frequently perceived screen time as negatively affecting their eyesight (61.8% vs. 48.8%, *p* < 0.001), time for physical activity and sports (40.0% vs. 33.2%, *p* = 0.022), time for live social contacts (31.1% vs. 21.2%, *p* < 0.001), and time for domestic or other duties (27.2% vs. 20.2%, *p* = 0.008). In contrast, foreign students more frequently reported adverse effects on their sleep time (72.6% vs. 61.8%, *p* < 0.001) and mental state (44.8% vs. 33.0%, *p* < 0.001) (Table 5). Sex-stratified analyses showed a similar pattern. Among male students, the only additional significant difference was that Bulgarian men more frequently reported a negative impact on time for live social contacts (28.7% vs. 17.5%, *p* = 0.007), whereas among female students, Bulgarian women more frequently reported adverse effects on time for domestic or other duties (25.6% vs. 16.7%, *p* = 0.006) and their eyesight (66.5% vs. 50.0%, *p* < 0.001). The greater perceived impact of screen time on sleep and mental state among foreign students remained evident in both sexes (Table 5).

**Table 4:** Frequency distribution of responses to the question “To which of the following do you think your screen time is harmful?” and comparative analyses of the two groups of students and the two sexes.^*^

Answers	*n*	%	Sp
Your sleep time	711	66.9	1.4
Your eyesight	592	55.7	1.5
Your mental state	410	38.6	1.5
Time for physical activity and sports	391	36.8	1.5
Time for live social contacts	281	26.4	1.4
Time for domestic or other duties	254	23.9	1.3
Time for your family	233	21.9	1.3
Time for work	177	16.7	1.1
I do not believe screen time does harm to any of the listed	41	3.9	0.6
Time for studying/scientific activity	10	0.9	0.3
Other	6	0.6	0.2
**Total number of responders**	1,063	100.0	

^*^The sum of the percentages exceeds 100, as some of the students indicated more than one answer.

**Table 5:** Comparative analysis of the two groups of students in total and stratified by sex according to the answers to the question “To which of the following do you think your screen time is harmful?”^*^

Answers	Bulgarian students		Foreign students		*p*
	*n*	%	*n*	%	
Time for physical activity and sports	225	40.0	166	33.2	**0.022**
Time for live social contacts	175	31.1	106	21.2	**< 0.001**
Your mental state	186	33.0	224	44.8	**< 0.001**
Your sleep time	348	61.8	363	72.6	**< 0.001**
Time for your family	135	24.0	98	19.6	0.084
Time for work	88	15.6	89	17.8	0.337
Time for domestic or other duties	153	27.2	101	20.2	**0.008**
Time for studying/scientific activity	290	51.5	250	50.0	0.826
Your eyesight	348	61.8	244	48.8	**< 0.001**
I do not believe screen time does harm to any of the listed	25	4.4	16	3.2	0.310
Other	2	0.4	4	0.8	0.395
Answers	Male Bulgarian students		Male foreign students		*p*
	*n*	%	*n*	%	
Time for physical activity and sports	89	39.9	62	32.0	0.094
Time for live social contacts	64	28.7	34	17.5	**0.007**
Your mental state	66	29.6	88	45.4	**< 0.001**
Your sleep time	134	60.1	142	73.2	0.005
Time for your family	55	24.7	45	23.2	0.721
Time for work	39	17.5	35	18.0	0.804
Time for domestic or other duties	66	29.6	50	25.8	0.388
Time for studying/scientific activity	116	52.0	94	48.5	0.476
Your eyesight	122	54.7	91	46.9	0.112
I do not believe screen time does harm to any of the listed	13	5.8	6	3.1	0.187
Other	2	0.9	1	0.5	0.629
Answers	Female Bulgarian students		Female foreign students		*p*
	*n*	%	*n*	%	
Time for physical activity and sports	136	40.0	104	34.0	0.115
Time for live social contacts	111	32.6	72	23.5	**0.010**
Your mental state	120	35.3	136	44.4	**0.018**
Your sleep time	214	62.9	221	72.2	**0.012**
Time for your family	80	23.5	53	17.3	0.052
Time for work	49	14.4	54	17.6	0.267
Time for domestic or other duties	87	25.6	51	16.7	**0.006**
Time for studying/scientific activity	174	51.2	156	51.0	0.960
Your eyesight	226	66.5	153	50.0	**< 0.001**
I do not believe screen time does harm to any of the listed	12	3.5	10	3.3	0.889
Other	0	0.0	3	1.0	0.065

^*^The sum of the percentages exceeds 100 as some of the students indicated more than one answer. The bold values indicate statistically significant *p*-values.

When comparing the two sexes irrespective of nationality, women more frequently reported that screen time negatively affected their eyesight than men (58.7% vs. 51.1%, *p* = 0.015), whereas men more often indicated a negative impact on time for domestic or other duties (27.8% vs. 21.4%, *p* = 0.017) (Table 6). Among Bulgarian students, women more frequently reported that screen time negatively affected their eyesight compared with men (66.5% vs. 54.7%, *p* = 0.005), while no other sex-related differences reached statistical significance. Among foreign students, men more frequently reported that screen time negatively affected time for domestic or other duties than women (25.8% vs. 16.7%, *p* = 0.014), whereas no significant sex differences were observed for the remaining responses.

**Table 6:** Comparative analysis of the two sexes according to the answers to the question “To which of the following do you think your screen time is harmful?”^*^

Answers	Men		Women		*p*
	*n*	%	*n*	%	
Time for physical activity and sports	151	36.2	240	37.2	0.742
Time for live social contacts	98	23.5	183	28.3	0.083
Your mental state	154	36.9	256	39.6	0.377
Your sleep time	276	66.2	435	67.3	0.710
Time for your family	100	24.0	133	20.6	0.191
Time for work	74	17.7	103	15.9	0.442
Time for domestic or other duties	116	27.8	138	21.4	**0.017**
Time for studying/scientific activity	210	50.4	330	51.1	0.824
Your eyesight	213	51.1	379	58.7	**0.015**
I do not believe screen time does harm to any of the listed	19	4.6	22	3.4	0.322
Other	3	0.7	3	0.5	0.675
Answers	Bulgarian male students		Bulgarian female students		*p*
	*n*	%	*n*	%	
Time for physical activity and sports	89	39.9	136	40.0	0.981
Time for live social contacts	64	28.7	111	32.6	0.328
Your mental state	66	29.6	120	35.3	0.160
Your sleep time	134	60.1	214	62.9	0.664
Time for your family	55	24.7	80	23.5	0.744
Time for work	39	17.5	49	14.4	0.322
Time for domestic or other duties	66	29.6	87	25.6	0.297
Time for studying/scientific activity	116	52.0	174	51.2	0.853
Your eyesight	122	54.7	226	66.5	**0.005**
I do not believe screen time does harm to any of the listed	13	5.8	12	3.5	0.194
Other	2	0.9	0	0.0	0.080
Answers	Foreign male students		Foreign female students		*p*
	*n*	%	*n*	%	
Time for physical activity and sports	62	32.0	104	34.0	0.644
Time for live social contacts	34	17.5	72	23.5	0.110
Your mental state	88	45.4	136	44.4	0.827
Your sleep time	142	73.2	221	72.2	0.807
Time for your family	45	23.2	53	17.3	0.106
Time for work	35	18.0	54	17.6	0.909
Time for domestic or other duties	50	25.8	51	16.7	**0.014**
Time for studying/scientific activity	94	48.5	156	51.0	0.586
Your eyesight	91	46.9	153	50.0	0.500
I do not believe screen time does harm to any of the listed	6	3.1	10	3.3	0.902
Other	1	0.5	3	1.0	0.543

^*^The sum of the percentages exceeds 100, as some of the students indicated more than one answer. The bold values indicate statistically significant *p*-values.

### Night sleep duration and bedtime

Data on nocturnal sleep duration were available for 1,061 (99.8%) participants. The mean nocturnal sleep duration was 6.68 ± 1.18 h, ranging from 0 to 11 h. Although the distribution was nearly symmetrical, it significantly deviated from normality (Kolmogorov–Smirnov test, *p* < 0.001), with a mode of 7 h. No significant differences in mean nocturnal sleep duration were observed between Bulgarian and foreign students, either overall or after stratification by sex. Likewise, no significant differences were found between men and women within either nationality group (Supplementary Table 3). Overall, more than half of the participants (57.2%) reported sleeping 7–9 h per night, while 42.0% reported sleeping < 7 h, and only 0.7% reported sleeping more than 9 h (Table 7). The distribution of sleep duration categories did not differ significantly between Bulgarian and foreign students, either in the overall sample or after stratification by sex (Supplementary Table 4). Similarly, no statistically significant differences were observed between male and female students in the overall cohort or within either nationality group.

**Table 7:** Frequency distribution of answers to the question “How many hours do you sleep on average at night?”^*^

Answers	*n*	%	Sp
Between 7 and 9 h	608	57.2	1.5
Below 7 h	446	42.0	1.5
Above 9 h	7	0.7	0.2
Invalid/extreme responses	2	0.2	0.1
**All**	1,063	100.0	

^*^Valid nocturnal sleep-duration data were available for 1.061 participants; two responses were classified as invalid/extreme and excluded from analyses involving sleep duration.

### Bedtime

Regarding usual bedtime, the largest proportion of participants reported going to bed between 11:00 p.m. and midnight (39.0%), followed by between midnight and 1:00 a.m. (24.9%) and between 10:00 and 11:00 p.m. (15.1%), whereas bedtime before 9:00 p.m. was reported least frequently (0.5%) (Table 8). To facilitate interpretation and comparison of bedtime patterns, the original nine response categories were consolidated into three clinically meaningful groups: before 10:00 p.m. (5.1%), between 10:00 p.m. and midnight (54.1%), and after midnight (40.8%). Comparison between Bulgarian and foreign students demonstrated significant differences in bedtime patterns. Bulgarian students more frequently reported going to bed between 10:00 p.m. and midnight than foreign students (60.4% vs. 47.0%, *p* < 0.001), whereas foreign students more frequently reported going to bed after midnight (47.2% vs. 35.2%, *p* < 0.001). These differences remained significant in both men and women (Table 9). No statistically significant sex-related differences in bedtime categories were identified in the overall sample or within either nationality group.

**Table 8:** Frequency distribution of respondents according to answers to the question “What time do you usually go to bed at night?”

Answers	*n*	%	Sp
Between 11 p.m. and midnight	415	39.0	1.5
Between midnight and 1 am	265	24.9	1.3
Between 10 p.m. and 11 p.m.	160	15.1	1.1
Between 1 and 2 a.m.	124	11.7	1.0
Between 9 and 10 p.m.	49	4.6	0.6
Between 2 and 3 a.m.	26	2.4	0.5
Between 3 and 4 a.m.	12	1.1	0.3
After 4 a.m	7	0.7	0.2
Before 9 p.m.	5	0.5	0.2
**Total number of responders**	1,063	100.0	

**Table 9:** Comparative analysis of the two groups of students and the two sexes according to the answers to the question “What time do you usually go to bed at night?”

Group	Answers	Bulgarian students		Foreign students		p
		*n*	%	*n*	%	
All (*n* = 1,063)	Before 10 p.m.	25	4.4	29	5.8	0.299
	Between 10 p.m. and midnight	340	60.4	235	47.0	**< 0.001**
	After midnight	198	35.2	236	47.2	**< 0.001**
Men (*n* = 417)	Before 10 p.m.	12	5.4	9	4.6	0.710
	Between 10 p.m. and midnight	124	55.6	85	43.8	**0.016**
	After midnight	87	39.0	100	51.5	**0.011**
Women (*n* = 646)	Before 10 p.m.	13	3.8	20	6.5	0.119
	Between 10 p.m. and midnight	216	63.5	150	49.0	**< 0.001**
	After midnight	111	32.6	136	44.4	**0.002**
Group	Answers	Men		Women		p
		*n*	%	*n*	%	
All (*n* = 1,063)	Before 10 p.m.	21	5.0	33	5.1	0.096
	Between 10 p.m. and midnight	209	50.1	366	56.7	
	After midnight	187	44.8	247	38.2	
Bulgarian Students (*n* = 563)	Before 10 p.m.	12	5.4	13	3.8	0.155
	Between 10 p.m. and midnight	124	55.6	216	63.5	
	After midnight	87	39.0	111	32.6	
Foreign Students (*n* = 500)	Before 10 p.m.	9	4.6	20	6.5	0.271
	Between 10 p.m. and midnight	85	43.8	150	49.0	
	After midnight	100	51.5	136	44.4	

The bold values indicate statistically significant *p*-values.

### Daytime sleep duration

Overall, 472 students (44.4%) reported no daytime sleep (0 min), while 588 (55.3%) reported some daytime sleep (>0 min). In the total sample, 21.1% reported daytime sleep of ≤ 30 min and 34.2% reported >30 min (Table 10). Among the 588 participants reporting any daytime sleep (>0 min), the mean duration was 72.47 ± 84.30 min (range: 1–540 min). The distribution was significantly non-normal (Kolmogorov–Smirnov test, *p* < 0.001), with a mode of 60 min. Foreign students reported significantly longer daytime sleep than Bulgarian students (86.42 ± 105.33 vs. 61.26 ± 60.34 min, *p* = 0.039). This difference remained significant among men (*p* = 0.032), but not among women (*p* = 0.330). No significant sex differences were observed within either nationality group (Supplementary Table 5).

**Table 10:** Frequency distribution and comparative analysis of the groups of students and the two sexes according to the answers to the question “How many minutes do you sleep on average during the day?”^*^

Answers	*n*		%		Sp	
0 min	472		44.4		1.5	
Up to 30 min	224		21.1		1.3	
Over 30 min	364		34.2		1.5	
Missing data	3		0.3		0.2	
**All**	1,063		100.0		0.0	
Group	Answers	Bulgarian students		Foreign students		*p*
		*n*	%	*n*	%	
All (*n* = 1,060)	0 min	237	42.1	235	47.3	0.196
	Up to 30 min	128	22.7	96	19.3	
	Over 30 min	198	35.2	166	33.4	
Men (*n* = 415)	0 min	92	41.3	106	55.2	**0.005**
	Up to 30 min	49	22.0	27	14.1	**0.038**
	Over 30 min	82	36.8	59	30.7	0.191
Women (*n* = 645)	0 min	145	42.6	129	42.3	0.958
	Up to 30 min	79	23.2	69	22.6	
	Over 30 min	116	34.1	107	35.1	
Group	Answers	Men		Women		*p*
		*n*	%	*n*	%	
All (*n* = 1,060)	0 min	198	47.7	274	42.5	0.128
	Up to 30 min	76	18.3	148	22.9	
	Over 30 min	141	34.0	223	34.6	
Bulgarian Students (*n* = 563)	0 min	92	41.3	145	42.6	0.820
	Up to 30 min	49	22.0	79	23.2	
	Over 30 min	82	36.8	116	34.1	
Foreign students (*n* = 497)	0 min	106	55.2	129	42.3	**0.005**
	Up to 30 min	27	14.1	69	22.6	**0.020**
	Over 30 min	59	30.7	107	35.1	0.312

^*^Daytime-sleep data were available for 1.060 participants; 3 responses were missing. The bold values indicate statistically significant *p*-values.

No significant differences in daytime sleep categories were observed between Bulgarian and foreign students overall. However, among men, foreign students more frequently reported not sleeping during the day (55.2% vs. 41.3%, *p* = 0.005), whereas Bulgarian men more often reported sleeping up to 30 min (22.0% vs. 14.1%, *p* = 0.038). Among foreign students, men more frequently no daytime sleep than women (55.2% vs. 42.3%, *p* = 0.005), while women more frequently reported sleeping up to 30 min (22.6% vs. 14.1%, *p* = 0.020).

### Correlation analysis between screen time and sleep characteristics

Correlation analysis demonstrated (Table 11) a significant association between screen time and sleep-related parameters, with the most consistent relationship observed for bedtime. Longer screen time was significantly associated with a later bedtime across all examined subgroups (*r* = 0.146–0.344), with a weak but significant correlation in the overall study population (*r* = 0.240; *p* < 0.01). The association was even more pronounced among alcohol abstainers, where the correlation reached a moderate level.

**Table 11:** Correlations between the reported screen time and nighttime sleep duration, daytime sleep duration, and bedtime hour in the overall study population and stratified by sex, study group, employment status, smoking status, alcohol consumption, and physical activity level.^*^

Sleep parameter	Screen time												
	Overall	Men	Women	Bulgarian students	Foreign students	Unemployed	Employed	Currently smoking	Currently non-smoking	Alcohol abstainers	Alcohol consumers	Not meeting physical activity recommen-dations	Meeting physical activity recommen-dations
Night sleep duration	−0.049	0.003	−0.084^*^	0.012	−0.129^**^	−0.059	−0.018	−0.048	−0.072	−0.154^**^	0.012	0.013	−0.069
Day sleep duration	0.101^**^	0.033	0.151^**^	0.164^**^	0.051	0.068	0.226^**^	0.092^**^	0.161^*^	0.050	0.132^**^	0.012	0.129^**^
Bedtime hour	0.240^**^	0.209^**^	0.255^**^	0.146^**^	0.268^**^	0.252^**^	0.200^**^	0.244^**^	0.248^**^	0.344^***^	0.181^**^	0.265^**^	0.228^**^

Values are Spearman's rank correlation coefficients (ρ). Pairwise deletion was used for missing data; therefore the number of participants included in each analysis varied according to the availability of valid data for both variables. For the overall analyses, *n* = 1,055 for screen time and nighttime sleep duration. *n* = 1,054 for screen time and daytime sleep duration and *n* = 1,057 for screen time and bedtime. ^*^p < 0.05; ^**^p < 0.01; ^***^p < 0.001.

The relationship between screen time and nighttime sleep duration was generally weak and non-significant in the total population (*r* = −0.049). However, significant negative correlations were observed for women (*r* = −0.084; *p* < 0.05), foreign students (*r* = −0.129; *p* < 0.01), and alcohol abstainers (*r* = −0.154; *p* < 0.01), suggesting a tendency toward shorter nighttime sleep with increased screen exposure in these groups.

Screen time showed a weak positive correlation with daytime sleep duration in the overall sample (*r* = 0.101; *p* < 0.01). Significant positive associations were observed among several subgroups, with correlation coefficients ranging from *r* = 0.092 to *r* = 0.226, including women (*r* = 0.151; *p* < 0.01), Bulgarian students (*r* = 0.164; *p* < 0.01), employed participants (*r* = 0.226; *p* < 0.01), currently smoking (*r* = 0.092; *p* < 0.01) and currently non-smoking participants (*r* = 0.161; *p* < 0.05), alcohol consumers (*r* = 0.132; *p* < 0.01), and physically active individuals (*r* = 0.129; *p* < 0.01).

## Discussion

### Screen time

The World Health Organization (WHO) provides specific recommendations regarding sedentary screen time primarily for children under 5 years, advising that screen exposure should be avoided in infants and limited to no more than 1 h per day among children aged 2–4 years (12). As far as adults and young adults are concerned, WHO emphasizes the importance of reducing sedentary behavior and replacing prolonged sitting time with physical activity; however, no specific upper limit for daily screen time has yet been established (16, 17). The absence of adult-specific screen time recommendations is particularly relevant for university students, among whom digital technologies are increasingly integrated into academic, professional, and social activities. Nevertheless, the absence of an established threshold should not be interpreted as an absence of risk as screen time is associated with various adverse effects on health as mentioned before. This issue is particularly relevant among medical students and professionals engaged in mentally demanding occupations, where prolonged screen exposure has become increasingly prevalent due to the extensive use of electronic resources, digital learning platforms and online communication (18, 19).

In our study population, medical students reported substantial daily screen exposure, with a mean screen time of 5.64 ± 2.81 h/day. Notably, only a small proportion of participants reported low screen exposure (< 2 h/day), whereas altogether more than one-fifth of students reported spending over 6 h/day in front of screens. The values we report are slightly lower compared to the 2024 values of screen time duration reported by Liebig et al. for medical students in Germany—7.7 h (19). In our study, the most frequently reported purposes of screen use were entertainment (78.3%) and studying/scientific activities (72.5%), demonstrating that screens serve simultaneously as essential educational tools and major sources of leisure engagement.

An important finding of our study was the significantly higher daily screen time reported by foreign students compared with Bulgarian students, with a difference of nearly 2 h per day. In addition, foreign students were disproportionately represented in the highest screen time categories, with approximately two-fold higher proportions reporting more than 6, 8, and 10 h of daily screen use. This difference remained consistent among both male and female participants. Additionally, no significant differences in screen exposure were observed between men and women within either student group, suggesting that factors related to the study environment and social context may have a greater influence on screen use patterns than sex alone.

Students appeared highly conscious of the adverse effects of screen time, with fewer than 4% reporting no perceived harm. Nevertheless, this high level of awareness contrasted with the substantial daily screen exposure, suggesting that knowledge alone may be insufficient to modify screen-related behaviors. Interestingly, Bulgarian students more frequently perceived adverse effects on eyesight, physical activity, face-to-face social interactions, and daily responsibilities and had a lower screen time duration. In contrast, foreign students, who reported nearly 2 h more screen time per day, more frequently perceived negative effects on sleep and mental health. One possible explanation is that greater awareness of the broader lifestyle consequences among Bulgarian students may contribute to more restrained screen use, whereas the higher exposure among foreign students may have reached a level at which consequences affecting sleep and psychological wellbeing become more apparent. However, given the cross-sectional nature of the study, these findings should be interpreted cautiously, and no causal or directional relationship can be established.

### Nighttime Sleep

Unlike many other health-related behaviors, such as physical activity, for which clear quantitative recommendations have been established, no specific World Health Organization recommendations currently state an optimal duration of sleep for healthy adults. The American Academy of Sleep Medicine recommends that adults should regularly obtain at least 7 h of sleep per night to promote optimal health and reduce adverse outcomes associated with insufficient sleep. Similarly, the National Sleep Foundation recommends 7–9 h of sleep per night for young adults aged 18–25 years and adults aged 26–64 years (20, 21). These recommendations are supported by evidence demonstrating that insufficient sleep negatively affects cognitive functioning. A meta-analysis of 61 studies showed that experimentally induced sleep restriction significantly impaired neurocognitive performance, with the most pronounced effects observed in executive function, sustained attention, and long-term memory. These findings are particularly relevant for medical students and other individuals engaged in cognitively demanding activities (22).

Beyond cognitive consequences, insufficient sleep has also been associated with a broad range of adverse physical health outcomes. A large systematic review and meta-analysis including more than 5 million participants from 153 prospective studies demonstrated that short sleep duration was associated with increased risks of mortality, diabetes mellitus, hypertension, cardiovascular diseases, coronary heart disease, and obesity (23). In our study, the mean nocturnal sleep duration was 6.68 ± 1.18 h, with 42.0% of participants reporting < 7 h of sleep per night, suggesting that insufficient sleep is common among medical students despite existing recommendations. Prolonged sleep duration has also been associated with adverse health outcomes. Large epidemiological studies have demonstrated a U-shaped relationship between sleep duration and health, with both short and long sleep durations being associated with increased risk of cardiovascular disease, stroke, and all-cause mortality (24, 25). In our study, prolonged sleep duration was uncommon, with only 0.7% of participants reporting more than 9 h of nocturnal sleep, limiting further interpretation of this sleep pattern. A recent large systematic review and meta-analysis (26) of 109 studies involving 59,427 medical students reported a pooled prevalence of poor sleep quality of 55.64% (95% CI, 51.45–59.74%), while 33.32% (95% CI, 26.52–40.91%) experienced excessive daytime sleepiness. Moreover, the pooled mean sleep duration was only 6.5 h per night, with the authors estimating that at least 30% of medical students sleep less than the recommended 7–9 h per night. These findings highlight that insufficient and disturbed sleep are widespread features of medical education across different countries and study settings.

Unlike sleep duration, for which some consensus recommendations exist, there is no universally accepted recommended bedtime hour for healthy adults. Current sleep guidelines instead emphasize maintaining a regular sleep schedule and obtaining sufficient sleep duration (20, 21). Nevertheless, growing evidence suggests that sleep timing may also represent an important determinant of health. In a large UK Biobank cohort study including more than 100,000 participants with objectively measured sleep onset timing using wrist accelerometry, Nikbakhtian et al. (27) demonstrated that sleep onset between 10:00 and 10:59 p.m. was associated with the lowest incidence of cardiovascular disease. Both earlier and later sleep onset, particularly sleep onset after midnight, were associated with higher cardiovascular risk, even after adjustment for sleep duration, sleep irregularity, and established cardiovascular risk factors. Although these findings do not establish causality, they suggest that delayed sleep timing may represent an additional marker of cardiovascular risk. In our study, delayed sleep time was common among medical students, with 40.8% reporting a usual bedtime after midnight and only 15.1% of participants reporting going to bed between 10:00 and 11:00 p.m. Foreign students were significantly more likely to report bedtime after midnight compared with Bulgarian students, and this difference remained significant in both sexes.

The difficulties of studying abroad may be partially reflected in the variations in screen time, sleep length, and bedtime among international students. Establishing new social and daily habits, communicating in a foreign language, and adjusting to a new academic and cultural setting can all provide significant challenges for foreign students. According to earlier studies (28, 29), these difficulties may cause acculturative stress in international students, although social support seems to be crucial in easing this stress. Furthermore, stress may be linked to lower sleep quality among international students, according to research comparing domestic and international university students. These factors may therefore contribute to differences in sleep duration, bedtime, and screen use, although they were not specifically assessed in the present study.

### Daytime sleep

We categorized daytime sleep into **≤30 min and** **>30 min** based on evidence that naps lasting longer than 30 min are more likely to be associated with **sleep inertia**—a transient state of reduced alertness and impaired cognitive performance immediately after awakening, often accompanied by grogginess and confusion. Distinguishing shorter from longer naps therefore provides a clinically meaningful classification, as shorter naps are less likely to result in these negative post-awakening effects and are generally considered more restorative (30). Among students who napped in our study, longer naps (>30 min) were more common than short naps ( ≤ 30 min), accounting for 61.9% and 38.1% of daytime sleepers, respectively. Although no significant differences in daytime sleep categories were observed between Bulgarian and foreign students overall, sex-specific analyses revealed distinct patterns. Foreign men were more likely than Bulgarian men to report no daytime sleep, whereas Bulgarian men more frequently reported short naps ( ≤ 30 min). Similarly, among foreign students, women were more likely than men to take short daytime naps, while men more frequently reported not napping. This finding is consistent with previous research among university students suggesting that women may experience greater daytime sleepiness and may be more likely to use daytime napping as a compensatory behavior. A recent study (31) of 757 university students found that women were more likely to nap and reported higher levels of stress and daytime sleepiness than men, with sex, perceived stress, and daytime sleepiness being associated with napping frequency.

### Correlations

Correlation analysis revealed that higher screen time was most consistently associated with a later bedtime with this association was observed across all examined subgroups. In contrast, the association between screen time and nighttime sleep duration was significant only in selected subgroups. The positive association between screen time and daytime sleep duration may indicate a compensatory response to a delayed nocturnal sleep, although the cross-sectional design does not allow conclusions regarding causality. Our findings are consistent with previous studies demonstrating that greater screen exposure is associated with delayed sleep timing among university students. In a study among German medical students, Liebig et al. (19) reported that higher leisure screen time was significantly associated with later bedtime (*r* = 0.213; *p* < 0.001), whereas the association with sleep duration was weaker and dependent on the type of screen exposure. Specifically, screen use related to leisure activities was primarily associated with delayed bedtime, while screen time related to study or work showed a tendency toward shorter sleep duration.

In another study by Liebig et al. (32), students reported approximately 7 h of daily screen time and 7.25 h of nocturnal sleep, whereas participants in our study reported a lower mean screen time of 5.64 ± 2.81 h per day and a shorter mean nocturnal sleep duration of 6.68 ± 1.18 h. The proportion of students sleeping < 7 h was also higher in our sample (42.0% vs. 23%). Despite these differences, both studies identified a significant association between greater screen exposure and later bedtime. Liebig et al. reported a correlation of *r* = 0.213 (*p* < 0.001) between leisure screen time and later bedtime, while our study demonstrated a similar, slightly stronger correlation (ρ = 0.240, *p* < 0.01). In contrast, the overall association between screen time and nighttime sleep duration was weak and not statistically significant in our study (ρ = −0.049), although a significant negative association was observed among foreign students (ρ = −0.129, *p* < 0.01). Similarly, in the German study, the relationship between screen time and sleep duration was weaker and depended on the purpose of screen use, with study- or work-related screen time showing an association with shorter sleep duration.

Overall, our findings highlight prolonged screen exposure as an emerging and potentially persistent behavioral risk factor associated with unfavorable sleep patterns among medical students. Unlike many other health-related risk behaviors, such as smoking or excessive alcohol consumption, simply emphasizing that screen time is harmful is unlikely to be sufficient, as digital technologies are now deeply integrated into modern education, professional activities, and social interactions. Similarly, insufficient and poorly timed sleep represents an important but often underestimated health concern among young adults, particularly those engaged in cognitively demanding professions. Given the established consequences of inadequate sleep for performance, mental health, and long-term cardiometabolic health, improving sleep-related behaviors should be considered an important preventive target. Further research should examine whether acculturation, language-related difficulties, social support, and other aspects of living abroad contribute to the differences observed between Bulgarian and foreign medical students.

### Limitations

This study has several limitations. The questionnaire was developed specifically for this study and was administered anonymously online; therefore, test–retest reliability could not be assessed. However, the questionnaire primarily assessed specific behavioral characteristics, such as screen time and sleep patterns, rather than a single underlying psychometric construct. The non-probability convenience sampling approach and voluntary participation may have introduced selection bias. The sample may not be fully representative of the broader medical student population, limiting the generalizability of the findings beyond the Medical University of Plovdiv. The single-institution study population may limit the generalizability of the findings. The cross-sectional design precludes establishing temporal or causal relationships between screen time and sleep-related outcomes. Although higher screen time was associated with later bedtime and, in some subgroups, shorter nighttime sleep, the direction of these associations cannot be determined. Greater screen exposure may contribute to delayed sleep, but conversely, students with later or disrupted sleep may engage in greater screen use during the evening or nighttime. Screen time and sleep parameters were self-reported and may be subject to recall bias. Although the anonymous nature of the survey may have reduced social desirability bias, objective measures were not incorporated into the study design. Therefore, some measurement error cannot be excluded, and the direction and magnitude of any potential bias cannot be reliably determined. Unmeasured factors, such as stress, academic workload, and mental health, may have influenced the observed associations. The timing of data collection, which spanned from May to December, may have introduced seasonal variation in studied patterns. Additionally, information on the use of potentially sleep-affecting beverages such as coffee, tea or energy drinks, was not collected and therefore could not be taken into account. The observational nature of the study does not permit complete exclusion of residual confounding.

### Conclusion

Our study demonstrates that prolonged screen exposure and suboptimal sleep patterns are common among medical students. Higher screen time was consistently associated with delayed bedtime, suggesting that excessive screen use may primarily affect sleep timing rather than sleep duration. Although digital technologies are essential components of contemporary medical education and daily life, their increasing integration highlights the need for strategies promoting healthier patterns.

## Data Availability

The raw data supporting the conclusions of this article will be made available by the authors, without undue reservation.
