# Supplementary material for: Patterns of screen use and their association with sleep among medical students: a cross-sectional study
Source: Front Public Health. 2026 Sep 18;14:1936680. doi: 10.3389/fpubh.2026.1936680 (PMC13630757; doi:10.3389/fpubh.2026.1936680)
Supplement: Supplementary file 1 [file Data_Sheet_1.pdf]

SUPPLEMENTARY CONTENT:

I. SCREEN TIME AND SLEEP QUESTIONS

1. How many hours is your screen* time per day?

** Screen time is the time spent in front of the screen of devices such as phones, computers, laptops, TV, video game console, and others.*

2. Most of your screen time is spent on which two of the following?

Multiple choice. Please select at most 2 options.

1) Studying/scientific process

2) Work

3) Seeking information for non-entertainment purposes, but not related to a studying/scientific process or work

4) Entertainment

3. To which of the following do you think your screen time is harmful?

Multiple choice.

1) Time for physical activity and sports

2) Time for live social contacts

3) Your mental state

4) Your sleep time

5) Time for your family

6) Time for work

7) Time for domestic or other duties

8) Time for studying/scientific activity

9) Your eyesight

10) I do not believe screen time does harm to any of the listed

4. How many HOURS do you sleep on average at night?

5. How many MINUTES do you sleep on average during the day?

6. What time do you usually go to bed at night?

1) before 9 p.m.

2) between 9 p.m. and 10 p.m.

3) between 10 p.m. and 11 p.m.

4) between 11 p.m. and midnight

5) between midnight and 1 a.m.

6) between 1 and 2 a.m.

7) between 2 and 3 a.m.

8) between 3 and 4 a.m.

9) after 4 a.m.

II. SUPPLEMENTARY TABLES:

Supplementary Table 1: Comparative analysis of the two groups of students according to the answers to the question "How many hours is your screen time per day?"*

Question	Group	Bulgarian Students			Foreign Students			P
		**n**	$\overline{X}$	SD	**n**	$\overline{X}$	SD	
How many hours is your screen time per day?	All	563	4.79	2.25	494	6.61	3.06	**<0.001**
	Men	223	4.99	2.30	192	6.73	3.06	**<0.001**
	Women	340	4.65	2.20	302	6.53	3.06	**<0.001**
	P=		0.088			0.370		

**Valid daily screen-time data were available for 1,057 of 1,063 participants.*

Supplementary Table 2. Comparison of daily screen time categories between male and female medical students, overall and by study group

Group	Screen time (hours)	Men		Women		p
		n	%	n	%	
All (n=1057)	Below 2	7	1.7	10	1.6	0.476
	2 - 4	146	35.2	249	38.8	
	4.01 - 6	129	31.1	210	32.7	
	6.01 - 8	77	18.6	93	14.5	
	8.01 - 10	30	7.2	48	7.5	
	Above 10	26	6.3	32	5.0	
Bulgarian Students (n=563)	Below 2	4	1.8	6	1.8	0.400
	2 - 4	105	47.1	176	51.8	
	4.01 - 6	69	30.9	112	32.9	
	6.01 - 8	30	13.5	29	8.5	
	8.01 - 10	8	3.6	11	3.2	
	Above 10	7	3.1	6	1.8	
Foreign Students (n=494)	Below 2	3	1.6	4	1.3	0.925
	2 - 4	41	21.4	73	24.2	
	4.01 - 6	60	31.3	98	32.5	
	6.01 - 8	47	24.5	64	21.2	
	8.01 - 10	22	11.5	37	12.3	
	Above 10	19	9.9	26	8.6	

**Valid daily screen-time data were available for 1,057 of 1,063 participants*

Supplementary Table 3: Comparative analysis of the two groups of students according to the answers to the question "How many hours do you sleep on average at night?"*

Question	Group	Bulgarian Students			Foreign Students			p
		n	$\bar{X}$	SD	n	$\bar{X}$	SD	
How many hours do you sleep on average at night?	All	561	6.66	1.19	500	6.69	1.18	0.638
	Men	222	6.74	1.29	194	6.64	1.18	0.374
	Women	339	6.62	1.11	306	6.73	1.18	0.184
	P=		0.073			0.633		

**Valid nighttime sleep-duration data were available for 1,061 of 1,063 participants*

Supplementary Table 4: Comparative analysis of the two groups of students according to the answers to the question "How many hours do you sleep on average at night?"

Group	Answers	Bulgarian Students		Foreign Students		P
		n	%	n	%	
All (n=1061)	Below 7 hours	239	42.6	207	41.4	0.916
	Between 7 and 9 hours	318	56.7	290	58.0	
	Above 9 hours	4	0.7	3	0.6	
Men (n=416)	Below 7 hours	82	36.9	82	42.3	0.273
	Between 7 and 9 hours	138	62.2	112	57.7	
	Above 9 hours	2	0.9	0	0.0	
Women (n=645)	Below 7 hours	157	46.3	125	40.8	0.310
	Between 7 and 9 hours	180	53.1	178	58.2	
	Above 9 hours	2	0.6	3	1.0	

Group	Answers	Men		Women		P
		n	%	n	%	
All (n=1061)	Below 7 hours	164	39.4	282	43.7	0.605
	Between 7 and 9 hours	250	60.1	358	55.5	
	Above 9 hours	2	0.5	5	0.8	
Bulgarian Students (n=561)	Below 7 hours	82	36.9	157	46.3	0.074
	Between 7 and 9 hours	138	62.2	180	53.1	
	Above 9 hours	2	0.9	2	0.6	
Foreign Students (n=500)	Below 7 hours	82	42.3	125	40.8	0.649
	Between 7 and 9 hours	112	57.7	178	58.2	
	Above 9 hours	0	0.0	3	1.0	

**Valid nighttime sleep-duration data were available for 1,061 of 1,063 participants*

Supplementary Table 5: Comparative analysis of the two groups of students and the two sexes according to the answers to the question "How many minutes do you sleep on average during the day?"*

Question	Group	Bulgarian Students			Foreign Students			P
		**n**	$\overline{X}$	SD	**n**	$\overline{X}$	SD	
How many minutes do you sleep during the day?	All	326	61.26	60.34	262	86.42	105.33	**0.039**
	Men	131	58.18	58.05	86	82.66	91.73	**0.032**
	Women	195	63.33	61.89	176	88.26	111.58	0.330
	P=		0.507			0.570		

Analyses of daytime sleep duration were restricted to participants who reported daytime sleep (>0 minutes; n = 588), including 326 Bulgarian and 262 foreign students. Participants reporting 0 minutes of daytime sleep and those with missing daytime-sleep data were not included in these analyses.
